# Measurement accuracy of CT systems: The importance of calibration phantoms

**DOI:** 10.1371/journal.pone.0332263

**Published:** 2025-09-25

**Authors:** Jan Scherberich, Anton G. Windfelder, Jessica Steinbart, Gabriele A. Krombach

**Affiliations:** 1 Department of Diagnostic and Interventional Radiology (Experimental Radiology), University Hospital Giessen, Giessen, Hesse, Germany; 2 Branch for Bioresources, Fraunhofer Institute for Molecular Biology and Applied Ecology IME, Giessen, Hesse, Germany; 3 Department of Radiology, Biruni University, Istanbul, Türkiye; Indiana University, UNITED STATES OF AMERICA

## Abstract

This study aims to evaluate the measurement accuracy of computed tomography (CT) systems, focusing on the necessity of using calibration phantoms for enhanced precision. Both clinical CT and micro-CT systems were evaluated using a specially designed two-ball phantom, which provides a reliable reference for spatial resolution and geometric accuracy. The study involved scanning the phantom with two micro-CT devices (the oversize micro-CT SkyScan 1173 and the high-resolution micro-CT SkyScan 1272) and a clinical CT device, a third-generation dual-source CT scanner (SOMATOM Force), measuring the distance between the centres of two ruby balls. The results showed significant differences in measurement accuracy between the devices. The high-resolution micro-CT provided the most consistent measurements with minimal variance, indicating its superiority in applications requiring high precision. In contrast, the oversize micro-CT exhibited larger errors, particularly at smaller voxel sizes, suggesting that internal calibration affected its accuracy. The dual source CT system had the smallest mean error but a larger standard deviation, indicating less consistency compared to micro-CT systems. Calibration with the two-ball phantom improved measurement accuracy across all devices. This improvement underscores the importance of using calibration phantoms to ensure accurate measurements, especially in fields that require high precision, such as clinical diagnostics and materials science. We concluded that routine calibration with phantoms is essential to achieve high measurement accuracy in CT imaging, thereby increasing the reliability of CT-based analyses in various disciplines.

## Introduction

Computed tomography (CT) represents a gold standard in imaging procedures for a multitude of clinical issues. The scan data obtained is often used to measure various structures non-invasively and in high resolution. In particular, micro-computed tomography (micro-CT) has demonstrated its value in a number of fields, offering the ability to visualise smaller samples in high resolution. Such common imaging indications include clinical applications such as bone or tooth analysis [1–7] and forensics [8], as well as also material sciences [9–15], mineral and soil sciences [16–19], life sciences [20–22] and the visualisation and measurement of various animal models [23–30].

In both types of scanners, namely the dual-source CT and the micro-CT, X-rays are emitted from a cathode in the beam direction towards a detector. The dimensions of the sample observed on the detector are contingent upon its position between the X-ray source and the detector itself. A series of projection images is captured from varying angles, which can then be used to generate cross-sectional images. The resulting size of the three-dimensional pixels (voxels) is typically determined by the calibration of the respective devices [31]. It is anticipated by device users that scans will be highly accurate, produced by factory-calibrated scanners without the use of further phantoms [25,32–34]. Typical pre-scan calibration steps only include stage alignment and flat-field corrections [35], however the usage of calibration objects is recommended [36,37].

**Fig 1:**
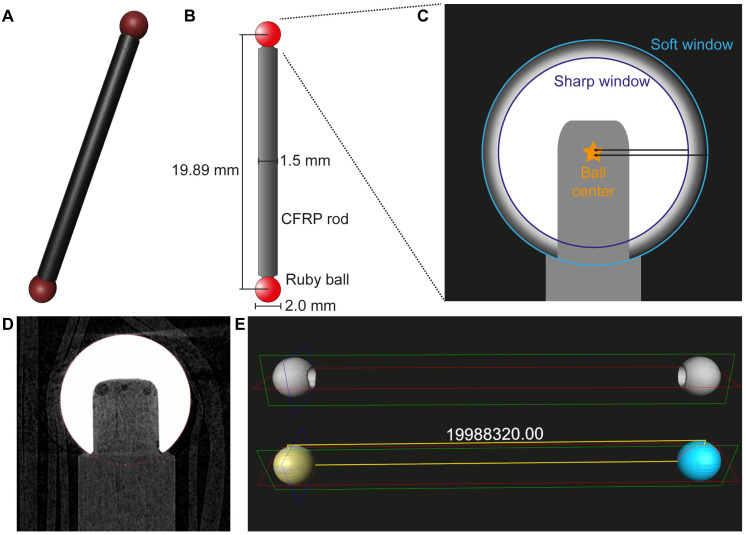
Two-ball phantom for CT calibration. (A) 3D rendering and (B) schematic image of a two-ball phantom. (C) For calibration purposes, the distance between the centre of the balls is used, as the outer edge may appear smaller or larger depending on the windowing in the image reconstruction (black arrows). The distance between the two spheres can be measured in 2D (D) or in 3D using matching spheres (E).

This raises a critical question regarding the metrological accuracy of measurements derived from CT or micro-CT scans. While users often trust the factory-calibrated settings of their devices, the actual accuracy may vary. It is therefore crucial to determine if further improvements are necessary through scans of a calibrated phantom. It is important to distinguish metrological accuracy—the correctness of a length measurement—from spatial resolution, which is the ability to distinguish fine details and is often tested with different tools like line-pair phantoms. The objective of this study is therefore to examine the measurement accuracy of different CT devices and to determine the necessity and effectiveness of using a calibration phantom to correct for systematic errors. The use of a ruby two-ball phantom, manufactured for the evaluation of spatial resolution and overall precision in CT imaging, allows for the empirical determination of the differences in performance of the systems (Fig 1). By establishing a fixed distance between the two spheres, the phantom serves as a reliable point of reference for calibrating the system’s spatial resolution and geometric precision. A different phantom, which measures the distance from edge to edge, would exhibit either softer or harder edges, which would depend on the windowing adjustment in the scan reconstruction (Fig 1B). As a result, the measured length would be different. This would also entail a change in the distance to be measured. Consequently, a two-ball phantom is employed. The distance between the centres of the balls is measured, as this distance is not affected by the windowing. It is anticipated that the highest level of measurement accuracy will be observed in micro-CT devices, while a greater degree of measurement inaccuracy will be evident in clinical CT, primarily due to the inferior resolution.

## Materials and methods

### Two-ball phantom

A two-ball phantom with a nominal 20 mm separation (ICT calibration sphere 20 mm 18040105, Goekeler Messtechnik GmbH, Lenningen, Germany) was utilised to assess the measurement accuracy of the CT systems. The phantom is composed of two high-precision ruby spheres, each with a diameter of 2 mm, connected by a CFRP rod (Fig 1). Ruby was chosen due to its ideal properties for X-ray tomography: it is extremely hard and chemically stable, and its high density and uniform composition provide excellent contrast and sharply defined edges in the resulting images. The definitive distance between the centres of the spheres is 19.89221 mm, a value certified by the German Accreditation Service (DAkkS; certificate: 49520) with a measurement uncertainty of only 0.0008 mm at a standard temperature of 20°C. This extremely low uncertainty confirms the phantom as a reliable gold standard for our study, ensuring that any measured deviations originate from the CT systems, not the phantom itself. The phantom was fixed in place using parafilm and styrofoam (for details see [38]).

### CT scanning and reconstruction

The two-ball phantom was scanned in two micro-CTs (the tabletop oversize micro-CT SkyScan 1173 & the tabletop high-resolution micro-CT SkyScan 1272, Bruker, Kontich, Belgium) and a clinical system, a third-generation dual-source CT scanner (SOMATOM Force, Siemens Healthineers, Forchheim, Germany) (Table 1). Scans were conducted at an ambient temperature of approximately 20°C to match the phantom’s certification conditions and minimize any potential for thermal expansion artifacts.

**Table 1:** Overview of the used CT systems.

	Oversize micro-CT	High-resolution micro-CT	Dual-source CT
Device name	SkyScan 1173	ScyScan 1272	SOMATOM Force
X-ray source	40 - 130 kV	40 - 100 kV	70 - 150 kV
Spot size	< 5 µm	< 5 µm	0.4 x 0.5 mm
Detector type	Flat panel sensor	Flat panel sensor	StellarInfinity
Detector size	112 x 112 mm, 2240 x 2240 pixel	36.9 x 36.9 mm 4096 x 4096 pixel	52.5 mm 1024 x 4096 pixel
Spatial resolution (10% MTF)	7 µm	< 5 µm	0.24 mm
Minimal voxel size	4 µm	< 0.45 µm	< 100 µm/ 400 µm

In each case, a central and a peripheral position were selected. For the micro-CT scans, the voxel size was divided into three positions, designated as near, middle, and far, by positioning the sample holder (Table 2). Each combination was measured seven times, resulting in a total of 98 scans. The X-ray projection images were saved in 16-bit TIFF (micro-CT) format or as DICOM (dual-source CT). Reconstructed cross-sectional images were stored in 8-bit PNG format using the software NRecon (NRecon 1.7.5.0, Bruker).

**Table 2:** Scanning and reconstruction parameters of the used CT systems. The dynamic range refers to the mapping of attenuation values to the 8-bit output scale during reconstruction of micro-CT scans.

	Oversize micro-CT	High-resolution micro-CT	Dual-source CT
Source voltage	110 kV	100 kV	80 kV, 150 kV
Source current	72 µA	100 µA	140 µA
Voxel size	11.021738 µm 14.932675 µm 19.909299 µm	6.000049 µm 13.000197 µm 22.999939 µm	97.6563 µm/ 400 µm
Exposure time	1100 ms	3212 ms	–
Rotation step	0.3°	0.3°	Continuous (0.6 mm)
Use 360 rotation	No (180°)	No (180°)	Yes
Frame averaging	4	4	–
Filter	Aluminum (1 mm)	Aluminum (1 mm)	Tin filter
Dynamic range/ windowing	0.00–0.03	0.00–0.03	W: 300, C: 40
Ring artifact correction factor	8	7	–
Beam hardening correction	30%	33%	–

### Analysis of scan data

The two-ball phantom scans were analysed in two and three dimensions. The 3D analysis was conducted using Amira-Avizo 2022.1 (Amira-Avizo 2022.1, ThermoFischer Scientific, Waltham, USA). The two ruby spheres were segmented using either a grey threshold of 80–255 (dual-source CT scans) or Otsu partitioning (micro-CT scans) and then marked with a fitted sphere. The centre-to-centre distances of the spheres were calculated for each scan (Fig 1E). For 2D evaluation, the scans were first aligned in the DataViewer software (DataViewer 1.7.0.1, Bruker) and an image in which both spheres were positioned at the centre was saved for further evaluation. The distances between the centres of the spheres in the 2D image were then measured in the Fiji/ImageJ software (Fiji 2.15.1, National Institutes of Health, USA) using a circle selection (Fig 1D) [39]. Furthermore, to assess the scan sharpness in the centre or at the edge of the device measurement field, the grey values were measured for each sphere using a line of 50 pixels (25 pixels for scans of the dual-source CT), which was drawn over the edge of the sphere to the centre in Fiji/ImageJ (n = 112). A fit curve (Rodbard) was created for this grey value line and its gradient was calculated in the grey value range between 60–140. Scans of the phantom made by the dual-source CT were measured in sagittal and coronal plane. For measurements in the axial plane the scans were resliced using Analyze (Analyze 14.0, AnalyzeDirect, Overland Park, United States) from 97x97x400 µm voxels to 97x97x97 voxels to be processed without distortion in the DataViewer.

### Statistical analysis

Statistical analysis was conducted using PAST software (version 4.16c, [40]). Given the non-normal distribution of the data, medians were compared using the Kruskal-Wallis test followed by Dunn’s post hoc test.

## Results

To determine the measuring accuracy of clinical and micro-CT systems a calibrated two-ball phantom was scanned in a central or peripheral position using near, middle and far distances to the X-ray source.

### Measurement accuracy

**Fig 2:**
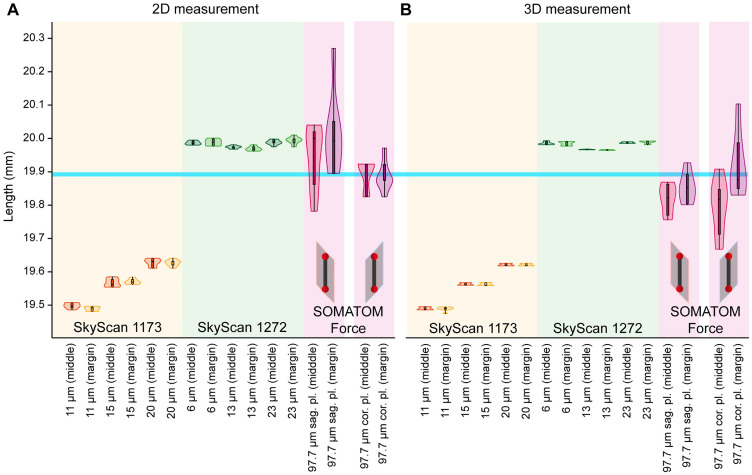
Measurement of phantom distance. CT scans of a calibrated 19.89221 mm (±0.0008 mm) two-ball phantom (blue line) were analysed in (A) 2D cross-sections and (B) 3D volume renderings and shown as violin plots (median, quartiles and distribution). Scans taken with the oversize micro-CT were below the correct phantom length and scans taken with the high-resolution micro-CT were above the correct phantom length. Dual-source CT scans were analysed in the sagittal plane in addition to the coronal plane.

With an average measurement difference of −0.329 mm (±0.056 mm) in the 2D image and −0.334 mm (±0.055 mm) in the 3D image, the oversize micro-CT had the largest error of the three devices compared to the phantom size of 19.89221 mm (±0.0008 mm measurement uncertainty). The measurement results of the oversize micro-CT were significantly lower compared to the high-resolution micro-CT (p: < 0.001) and the dual-source CT (p: < 0.001). However, measurements of the phantom with the high-resolution micro-CT had a lower measurement deviation of 0.091 mm (±0.012 mm) in the 2D image and 0.088 mm (±0.011 mm) above the expected value. Measurements using the dual-source CT scans had an average deviation of 0.094 mm (±0.116 mm) in the 2D image and −0.045 mm (±0.111 mm) in the 3D image, but also the largest standard deviation of the measured values (Fig 2). The most accurate measurement of all device/position combinations was the 2D measurement of the dual-source CT (centre position, coronal plane) with a distance of 19.895 mm (±0.047 mm), corresponding to an error of 0.013%. In contrast, the largest error of 2.02% was found with the oversize micro-CT (at 11 µm voxel size, positioned at the edge of the field of view). Varying the phantom position between the centre and the margin of the measuring field did not result in significant differences in the measurement of the sphere distance in any device. Changing the sample holder position (near, centre and far from the X-ray source) also showed no significant differences in any of the devices tested. However, there are clear increments between the sample holder distances, especially in the oversize micro-CT (2D measurement in the central position: 19.499 ± 0.008 mm in the near range, 19.567 ± 0.012 mm in the middle range and 19.628 ± 0.012 mm in the far range, Fig 2). The results are summarized in Table 3.

**Table 3:** Results of the measured scan accuracy.

Device	Voxel size (µm)	Position	Dataset axis	Measurement type	Average result (mm)	Absolute error (mm)	Percentual error (%)
Oversize micro-CT	11	center	axial	2D	19.499 ± 0.008	0.393	−1.98
Oversize micro-CT	11	margin	axial	2D	19.490 ± 0.007	0.403	−2.02
Oversize micro-CT	15	center	axial	2D	19.567 ± 0.012	0.325	−1.63
Oversize micro-CT	15	margin	axial	2D	19.570 ± 0.008	0.322	−1.62
Oversize micro-CT	20	center	axial	2D	19.628 ± 0.012	0.264	−1.33
Oversize micro-CT	20	margin	axial	2D	19.626 ± 0.010	0.266	−1.34
High-res. micro-CT	6	center	axial	2D	19.987 ± 0.005	−0.095	0.48
High-res. micro-CT	6	margin	axial	2D	19.988 ± 0.010	−0.096	0.48
High-res. micro-CT	13	center	axial	2D	19.974 ± 0.004	−0.082	0.41
High-res. micro-CT	13	margin	axial	2D	19.969 ± 0.007	−0.077	0.39
High-res. micro-CT	23	center	axial	2D	19.989 ± 0.008	−0.096	0.49
High-res. micro-CT	23	margin	axial	2D	19.994 ± 0.011	−0.102	0.51
Dual-source CT	97.7/400	center	axial	2D	20.048 ± 0.084	−0.155	0.78
Dual-source CT	97.7/400	margin	axial	2D	20.131 ± 0.047	−0.239	1.20
Dual-source CT	97.7/400	center	sagittal	2D	19.943 ± 0.097	−0.051	0.26
Dual-source CT	97.7/400	margin	sagittal	2D	20.014 ± 0.127	−0.122	0.61
Dual-source CT	97.7/400	center	coronal	2D	19.895 ± 0.047	−0.002	0.01
Dual-source CT	97.7/400	margin	coronal	2D	19.888 ± 0.046	0.005	−0.02
Oversize micro-CT	11	center	axial	3D	19.491 ± 0.005	0.401	−2.02
Oversize micro-CT	11	margin	axial	3D	19.489 ± 0.007	0.403	−2.03
Oversize micro-CT	15	center	axial	3D	19.563 ± 0.004	0.329	−1.65
Oversize micro-CT	15	margin	axial	3D	19.563 ± 0.005	0.329	−1.65
Oversize micro-CT	20	center	axial	3D	19.622 ± 0.003	0.270	−1.36
Oversize micro-CT	20	margin	axial	3D	19.622 ± 0.004	0.271	−1.36
High-res. micro-CT	6	center	axial	3D	19.985 ± 0.005	−0.093	0.47
High-res. micro-CT	6	margin	axial	3D	19.985 ± 0.007	−0.093	0.47
High-res. micro-CT	13	center	axial	3D	19.967 ± 0.000	−0.074	0.37
High-res. micro-CT	13	margin	axial	3D	19.965 ± 0.001	−0.073	0.39
High-res. micro-CT	23	center	axial	3D	19.987 ± 0.002	−0.095	0.48
High-res. micro-CT	23	margin	axial	3D	19.989 ± 0.005	−0.096	0.49
Dual-source CT	97.7/400	center	axial	3D	19.937 ± 0.046	−0.045	0.23
Dual-source CT	97.7/400	margin	axial	3D	20.080 ± 0.014	−0.188	0.94
Dual-source CT	97.7/400	center	sagittal	3D	19.820 ± 0.044	0.072	−0.36
Dual-source CT	97.7/400	margin	sagittal	3D	19.853 ± 0.045	0.039	−0.20
Dual-source CT	97.7/400	center	coronal	3D	19.797 ± 0.083	0.095	−0.48
Dual-source CT	97.7/400	margin	coronal	3D	19.920 ± 0.097	−0.027	0.14
Summary of scans in all positions:							
Oversize micro-CT	11-20	all	axial	2D	19.563 ± 0.056	0.329	−1.65
High-res. micro-CT	6-23	all	axial	2D	19.984 ± 0.012	−0.091	0.46
Dual-source CT	97.7/400	all	all	2D	19.986 ± 0.116	−0.094	0.47
Oversize micro-CT	11-20	all	axial	3D	19.558 ± 0.055	0.334	−1.68
High-res. micro-CT	6-23	all	axial	3D	19.980 ± 0.011	−0.088	0.44
Dual-source CT	97.7/400	all	all	3D	19.901 ± 0.111	−0.009	0.05

**Fig 3:**
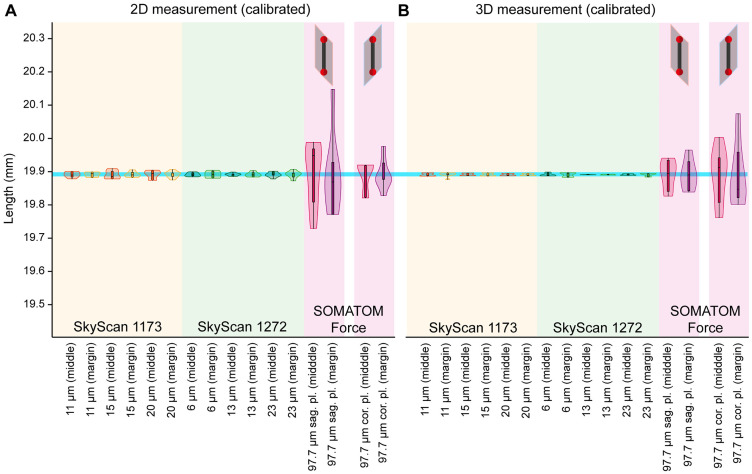
Corrected measurements of phantom distance after calibration. Measurement results after adjustment of the mean values to the size of the calibrated two-ball phantom of 19.89221 mm (blue line) in (A) 2D cross-sections and (B) 3D volume renderings. The corrected results show a good representation of the actual phantom size. Measurement of scans taken with the SOMATOM Force showed a greater scatter of data compared to the micro-CTs.

By calibrating the CT scan data with the two-ball phantom for each position and distance setting using the mean values, the measurement result can be improved (Fig 3). After adjusting the averages to the size of the phantom (19.89221 mm), very accurate measurement values were obtained for the micro-CTs. The measured values of the oversize micro-CT were in the range between 19.877 mm and 19.899 mm (0.021 mm difference) and those of the high-resolution micro-CT were in the range between 19.884 mm and 19.900 mm (0.016 mm difference) in the 3D image. The range of measurements for the dual-source CT scans was between 19.763 mm and 20.076 mm (0.313 mm difference) and was therefore larger than for micro-CT. The range of the measurements for the 2D image showed a similar picture but was slightly higher than that for the 3D image. For the oversize micro-CT, the values were in the range between 19.875 mm and 19.910 mm (0.035 mm difference), for the high-resolution micro-CT in the range between 19.873 mm and 19.908 mm (0.034 mm difference) and for the dual-source CT in the range between 19.732 mm and 20.146 mm (0.414 mm difference).

**Fig 4:**
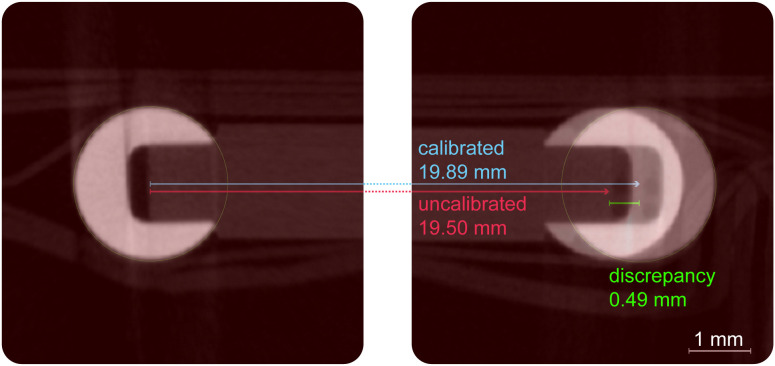
Visual comparison of uncalibrated and calibrated measurements. An overlay on a 2D slice from the oversize micro-CT shows the uncalibrated measured distance (red line and overlay), which is visibly shorter than the actual center-to-center distance. After applying the correction factor derived from the phantom, the calibrated measurement (blue line) accurately aligns with the centers of the two ruby spheres, demonstrating the effectiveness of the calibration.

To visually demonstrate the impact of calibration, the uncalibrated measurement error can be compared directly against the corrected measurement. After applying the calculated correction factor, the adjusted measurement (Fig 4) accurately reflects the true phantom geometry.

### Scan sharpness

**Fig 5:**
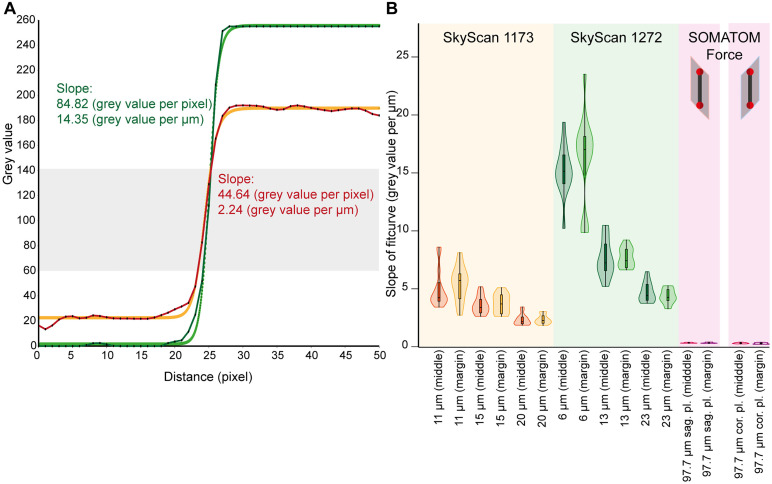
Phantom edge profile curves. A profile line of 50 pixels (25 pixels for SOMATOM Force scans) was placed over the outer edge of the ruby ball in each CT scan in the 2D view to determine the gradient of the grey values. (A) The slope of the fit curves (Rodbard fit) with a grey value between 60 and 140 (grey box) was determined for the profile lines and shown as an example for a scan in the high-resolution micro-CT (11 µm voxel size, central position) in red and the oversize micro-CT (20 µm voxel size, central position) in green. (B) Comparison of the slope of the profile lines (B). In the micro-CTs, scans with smaller voxel sizes have steeper profile lines. The steepest profile lines were measured in the high-resolution micro-CT scans.

**Fig 6:**
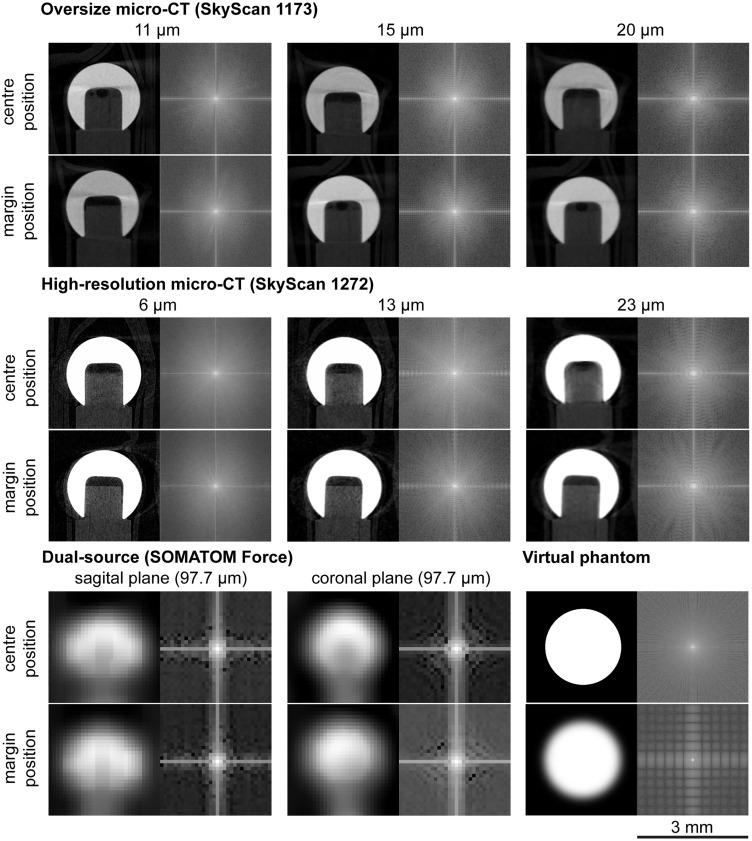
Exemplary CT scans cropped to one ruby ball. A cross-sectional view of the top ruby sphere of the phantom is presented in the 2D view with differing scan parameters (left) and the corresponding FFT conversion (right). Positions and voxel sizes were varied. For better interpretation, a computer-generated virtual phantom was added as an idealized reference case (bottom right image pair). This virtual image was created with a defined, slight blur at the edge to serve as a benchmark. The FFT of this virtual phantom produces a characteristic checkered pattern, which serves as a reference for how a blurred edge is represented in the frequency domain. In comparison, the FFTs of the real scans with larger voxel sizes (e.g., 23 µm) and consequently lower edge sharpness show ring-shaped gradations, indicating greater image blur. Scale: 3 mm.

In each CT scan, a profile line was placed over the outer edge of the ruby spheres in the 2D view. The slope of the profile line (Fig 5A) corresponds to the transition of the image from the background (black) to the radiopaque ruby ball (white). A decrease in the clarity of the edge of the phantom results in a corresponding decrease in the gradient of the profile line (Figs 5 and 6, S1 Fig). No significant differences were identified between the phantom positions (centre, margin) and the various measurement distances (near, middle and far) for scans conducted with the oversize micro-CT and the dual-source CT. Scans with the high-resolution micro-CT were significantly sharper in the near position (6 µm voxel size) (p central/margin: 0.003/0.001) than in the far position (23 µm voxel size). The scans obtained with the high-resolution micro-CT exhibited the greatest gradient of the profile line in the near position (central: 15.21 ± 2.12 grey values/µm, margin: 16.35 ± 3.82 grey values/µm). The scans performed on the dual-source CT exhibited the lowest gradient of the profile line, with values of 0.34 ± 0.05 grey values/µm for the central coronal scan and 0.29 ± 0.07 grey values/µm for the outer coronal scan. The differences in quality of the various voxel sizes are also evident in the FFT conversion of the respective sphere images (Fig 6). At far measurement positions, clear ring-shaped gradations are visible in the FFT display, which are caused by softer image edges.

## Discussion

In this study, the measurement accuracy of CT systems using a two-ball phantom is compared to assess whether it is necessary to check measurements with these devices by means of a further calibration scan with a phantom. While factory-calibrated settings provide a baseline accuracy, additional calibration with a phantom, such as the two-ball phantom used in this study, can significantly improve measurement precision. This is particularly crucial for applications requiring high accuracy, such as in clinical diagnostics, biological research and material sciences.

### Accuracy of the CT systems

Micro-CT systems are renowned for their high resolution, which is vital for comprehensive imaging of minute structures. The findings of this study demonstrate that the high-resolution micro-CT yielded the most uniform results with minimal variance, a conclusion supported by other research that underscores the superiority of micro-CT in visualising intricate details. [21]. This consistency is essential for applications that are dependent on high precision.

It was surprising to find that measurement errors of up to 2% (~0.3 mm below the expected value) could occur depending on the distance of the phantom with the oversize micro-CT. The results demonstrated that the errors were not randomly distributed but rather exhibited consistency, albeit dependent on the voxel size utilized. In this context, the smallest voxel size (best resolution) exhibited the greatest measurement error, while the largest voxel size (poorer resolution) demonstrated the most accurate measurement. This stepwise gradation of the measurement results is likely due to the internal calibration of the device, which estimates the size of the projected image on the detector. The oversize micro-CT, although less accurate than the high-resolution micro-CT, still provided reliable measurements within a narrow range after calibration.

Clinical CT systems, such as the dual-source CT, are designed to facilitate imaging of larger structures, including the human body. These systems typically have larger voxel sizes (e.g., 97.7 µm), which limits their resolution and precision in comparison to micro-CT systems. Notwithstanding, the dual-source CT exhibited a small average deviation of 0.094 ± 0.116 mm in 2D and 0.045 ± 0.111 mm in 3D images in comparison to micro-CT systems. However, this was accompanied by a larger standard deviation, indicating less consistency. The variability in measurement accuracy observed in the dual-source CT is consistent with the findings of Saxena et al. [41], who noted that clinical CT protocols can have difficulty discriminating fine details, such as nasal cartilage boundaries, in comparison to micro-CT. Nevertheless, the measurements of scans with the dual-source CT are remarkably accurate. This is also in line with measurements by Windfelder et al., who were able to measure and distinguish different pore sizes starting from 0.6 mm with an increment of 0.2 mm very consistently in a Derenzo phantom [23] in the same system.

### Recalibration of the factory settings

Systematic errors in CT measurements can arise from various sources, including inaccuracies in the measurement of distances between the X-ray source and the sample or detector. The utilisation of two-ball phantoms for the calibration of length measurements in CT systems, both dual-source CT and micro-CT, is a crucial practice to ensure the accuracy and precision of measurements. Calibration with a two-ball phantom is an important step in achieving high accuracy in CT measurements, as it provides a reliable reference for spatial resolution and geometric accuracy. Zemek et al. [31,42] constructed a self-designed two-ball phantom comparable to the phantom employed in this study, albeit with 0.3 mm ruby spheres, for the calibration of minute field of views in micro-CT scanners. The measurement of center-to-center distances between two spheres is minimally affected by edge detection and beam hardening, making it a robust method for voxel size calibration [43]. This is particularly important in micro-CT systems, where even small deviations can significantly impact measurement accuracy due to the high resolution involved. Once the error factor (the average measurement deviation against the phantom length of 19.89221 mm) had been taken into account, it was found that all devices were capable of measuring with great precision (Fig 3). (The standard deviation of the micro-CT was found to be no greater than 0.005 mm, while that of the dual-source CT was 0.067 mm in relation to the expected value).

### Influence of the phantom position on the resolution

The sharpness of edges in CT phantoms is of paramount importance for accurate imaging and analysis. It is indicated that the modulation transfer function (MTF) varies significantly across the field of view (FOV), suggesting that edges may indeed be sharper in the center compared to the periphery. MTF measurements of Sari et al. [44] showed a sharpness decrease as the FOV increases, with a notable difference in MTF values (10% and 50%) between smaller and larger FOVs, indicating sharper edges at the center. The study highlights that noise levels decrease with larger FOVs, which may contribute to perceived edge sharpness [44]. The profile lines across the edges of the phantom in this study demonstrated no statistically significant differences in their slope when positioned centrally or at the margin. A smaller voxel size, on the other hand, led to a significantly steeper slope of the profile lines. In contrast, a reduction in voxel size resulted in a markedly more pronounced slope of the profile lines. Consequently, in the context of micro-CTs, the voxel size appears to exert a greater influence on image sharpness than the positioning within the central field of view.

### Sources of measurement error and the role of calibration

Our results reveal that while all CT systems provide solid measurements, their inherent accuracy and precision vary significantly. Systematic errors, as seen prominently in the oversize micro-CT, can arise from multiple sources. The stepwise gradation of error with changing voxel size in the oversize micro-CT strongly suggests an inaccuracy in the system’s internal geometric calibration model, which estimates object size based on the projection geometry. This model may be less robust when extrapolating across the full range of possible sample magnifications. Crucially, our study demonstrates that such systematic, device-specific errors can be effectively corrected by applying a correction factor derived from a simple phantom scan.

The higher variability (i.e., larger standard deviation) observed in the dual-source clinical CT has different origins. It is primarily attributable to its much larger voxel size (e.g., 97.7 µm x 97.7 x 400 µm) compared to the micro-CT systems. These large voxels lead to more significant partial volume effects at the curved surface of the spheres, making the precise determination of the sphere’s geometric center less consistent across repeated measurements.

Beyond voxel size, the fundamental differences in hardware and software between the micro-CT systems and the clinical dual-source CT significantly contribute to the observed variations in measurement accuracy. The system components are optimized for vastly different applications. The micro-CT systems utilize high-density flat-panel sensors (e.g., 4096x4096 pixels) paired with a very small X-ray focal spot size (< 5 µm), which is fundamental for achieving high intrinsic spatial resolution. In contrast, the clinical SOMATOM Force detector (StellarInfinity) is optimized for rapid, low-dose acquisitions over a large anatomical field of view, which typically involves larger detector elements. This inherently limits its ability to resolve fine details with the same fidelity as a micro-CT detector. The reconstruction parameters also diverge based on their primary goal. The clinical CT employed a tin filter for spectral shaping, a technique used to optimize image quality and dose in advanced clinical protocols like dual-energy imaging. Such spectral filtering, combined with the sophisticated iterative reconstruction algorithms common in modern clinical scanners, is designed to manage noise and enhance diagnostic contrast in patient scans. This approach differs significantly from the simpler aluminum filtration and the cone-beam reconstruction algorithms (e.g., NRecon software) used for the micro-CTs, which are primarily aimed at achieving high geometric fidelity. Collectively, these differences in detector technology, X-ray spot size, reconstruction strategy, and acquisition geometry explain why the micro-CT systems, while exhibiting systematic errors that necessitate calibration, offer higher intrinsic precision for metrological tasks. The clinical CT, on the other hand, is optimized for patient safety, speed, and diagnostic image quality over ultimate measurement precision.

It is also important to acknowledge the limitations of the two-ball phantom used in this study. While excellent for calibrating linear distance and voxel size, it does not assess more complex, non-linear geometric distortions across the field of view, such as barrel or pincushion distortion. For such comprehensive geometric characterization, alternative phantoms like 3D grid phantoms would be required. Nevertheless, for a vast number of applications based on linear measurements, the two-ball phantom provides a robust, accessible, and highly effective method for accuracy correction.

## Conclusion

In conclusion, this study demonstrates that relying solely on factory calibrations for CT and micro-CT systems can lead to significant and systematic measurement errors. The necessity of user-performed calibration was confirmed, as applying a correction factor derived from a simple two-ball phantom dramatically improved measurement accuracy across all tested devices. Our findings show that the high-resolution micro-CT provides the most consistent measurements, making it superior for applications requiring high precision. For micro-CT measurements in particular, where errors can be dependent on the voxel size setting, probes should ideally be measured at a consistent voxel size, and a corresponding correction factor from a phantom scan should be applied for each measurement series. However, even less precise systems can yield highly accurate data once calibrated. Therefore, we conclude that the routine use of calibration phantoms is an essential and effective procedure to ensure measurement accuracy. We recommend that such a calibration be performed regularly (e.g., quarterly) and after any system maintenance or software update to guarantee the reliability of CT-based metrology in both research and clinical applications.

## Supporting information

S1 Fig Phantom edge profile curves in pixel. A profile line of 50 pixels (25 pixels for scans of the SOMATOM Force) was placed over the outer edge of the ruby spheres in each CT scan in the 2D view. This was done to determine the gradient of the grey values. The slope of the fit curves (Rodbard fit) was determined for the profile lines and compared with each other. The steepest profile lines were measured in the scans of the high-resolution micro-CT.
(TIF)

## References

[1] Khoury BM, Bigelow EMR, Smith LM, Schlecht SH, Scheller EL, Andarawis-Puri N, et al. The use of nano-computed tomography to enhance musculoskeletal research. Connect Tissue Res. 2015;56(2):106–19. doi: 10.3109/03008207.2015.1005211 25646568 PMC4755519

[2] Renders GAP, Mulder L, Lin AS, Langenbach GEJ, Koolstra JH, Guldberg RE, et al. Contrast-enhanced microCT (EPIC-μCT) ex vivo applied to the mouse and human jaw joint. Dentomaxillofac Radiol. 2014;43(2):20130098. doi: 10.1259/dmfr.20130098 24353248 PMC4064618

[3] Kerckhofs G, Sainz J, Wevers M, Van de Putte T, Schrooten J. Contrast-enhanced nanofocus computed tomography images the cartilage subtissue architecture in three dimensions. Eur Cell Mater. 2013;25:179–89. doi: 10.22203/ecm.v025a13 23389752

[4] Bouxsein ML, Boyd SK, Christiansen BA, Guldberg RE, Jepsen KJ, Müller R. Guidelines for assessment of bone microstructure in rodents using micro-computed tomography. J Bone Miner Res. 2010;25(7):1468–86. doi: 10.1002/jbmr.141 20533309

[5] Sasov A, Liu X, Salmon PL. Compensation of mechanical inaccuracies in micro-CT and nano-CT. In: Stock SR, editor. Developments in X-Ray Tomography VI. SPIE; 2008. p. 70781C.

[6] Schneider P, Stauber M, Voide R, Stampanoni M, Donahue LR, Müller R. Ultrastructural properties in cortical bone vary greatly in two inbred strains of mice as assessed by synchrotron light based micro- and nano-CT. J Bone Miner Res. 2007;22(10):1557–70. doi: 10.1359/jbmr.070703 17605631

[7] Schulz-Weidner N, Wang J, Steinbart J, Windfelder AG, Krombach GA, Krämer N, et al. Evaluation of mechanical versus manual root canal preparation in primary molars-a comparative in vitro study. J Clin Med. 2023;12(24):7718. doi: 10.3390/jcm12247718 38137787 PMC10743663

[8] Franchetti G, Viel G, Fais P, Fichera G, Cecchin D, Cecchetto G, et al. Forensic applications of micro-computed tomography: a systematic review. Clin Transl Imaging. 2022;10(6):597–610. doi: 10.1007/s40336-022-00510-y

[9] Gao Y, Hu W, Xin S, Sun L. A review of applications of CT imaging on fiber reinforced composites. J Compos Mater. 2021;56(1):133–64. doi: 10.1177/00219983211050705

[10] Vásárhelyi L, Kónya Z, Kukovecz Á, Vajtai R. Microcomputed tomography–based characterization of advanced materials: a review. Mater Today Adv. 2020;8:100084. doi: 10.1016/j.mtadv.2020.100084

[11] Du Z, Hu Y, Ali Buttar N, Mahmood A. X-ray computed tomography for quality inspection of agricultural products: a review. Food Sci Nutr. 2019;7(10):3146–60. doi: 10.1002/fsn3.1179 31660129 PMC6804772

[12] Reedy CL, Reedy CL. High-resolution micro-CT with 3D image analysis for porosity characterization of historic bricks. Herit Sci. 2022;10(1). doi: 10.1186/s40494-022-00723-4

[13] Scharf J, Chouchane M, Finegan DP, Lu B, Redquest C, Kim M-C, et al. Bridging nano- and microscale X-ray tomography for battery research by leveraging artificial intelligence. Nat Nanotechnol. 2022;17(5):446–59. doi: 10.1038/s41565-022-01081-9 35414116

[14] Khosravani MR, Reinicke T. On the use of X-ray computed tomography in assessment of 3D-printed components. J Nondestruct Eval. 2020;39(4). doi: 10.1007/s10921-020-00721-1

[15] Jago KLG. X-ray computed microtomography of rubber. Rubber Chem Technol. 2012;85(3):387–407. doi: 10.5254/rct.12.87985

[16] Guntoro P, Ghorbani Y, Koch P-H, Rosenkranz J. X-ray microcomputed tomography (µCT) for mineral characterization: a review of data analysis methods. Minerals. 2019;9(3):183. doi: 10.3390/min9030183

[17] Singh N, Kumar S, Udawatta RP, Anderson SH, de Jonge LW, Katuwal S. X-ray micro-computed tomography characterized soil pore network as influenced by long-term application of manure and fertilizer. Geoderma. 2021;385:114872. doi: 10.1016/j.geoderma.2020.114872

[18] Kumi F, Hanping M, Jianping H, Ullah I. Review of applying X-ray computed tomography for imaging soil-root physical and biological processes. Int J Agric Biol Eng. 2015;8:1–14. doi: 10.3965/j.ijabe.20150805.1490

[19] Taina IA, Heck RJ, Elliot TR. Application of X-ray computed tomography to soil science: a literature review. Can J Soil Sci. 2008;88(1):1–19. doi: 10.4141/cjss06027

[20] Rawson SD, Maksimcuka J, Withers PJ, Cartmell SH. X-ray computed tomography in life sciences. BMC Biol. 2020;18(1):21. doi: 10.1186/s12915-020-0753-2 32103752 PMC7045626

[21] Keklikoglou K, Arvanitidis C, Chatzigeorgiou G, Chatzinikolaou E, Karagiannidis E, Koletsa T, et al. Micro-CT for biological and biomedical studies: a comparison of imaging techniques. J Imaging. 2021;7(9):172. doi: 10.3390/jimaging7090172 34564098 PMC8470083

[22] du Plessis A, Yadroitsev I, Yadroitsava I, Le Roux SG. X-Ray microcomputed tomography in additive manufacturing: a review of the current technology and applications. 3D Print Addit Manuf. 2018;5(3):227–47. doi: 10.1089/3dp.2018.0060

[23] Windfelder AG, Müller FHH, Mc Larney B, Hentschel M, Böhringer AC, von Bredow C-R, et al. High-throughput screening of caterpillars as a platform to study host-microbe interactions and enteric immunity. Nat Commun. 2022;13(1):7216. doi: 10.1038/s41467-022-34865-7 36433960 PMC9700799

[24] Scherberich J, Taszus R, Stoessel A, Nowotny M. Comparative micromechanics of bushcricket ears with and without a specialized auditory fovea region in the crista acustica. Proc Biol Sci. 2020;287(1929):20200909. doi: 10.1098/rspb.2020.0909 32576108 PMC7329045

[25] Raś M, Iwan D, Kamiński MJ. The tracheal system in post-embryonic development of holometabolous insects: a case study using the mealworm beetle. J Anat. 2018;232(6):997–1015. doi: 10.1111/joa.12808 29574917 PMC5980188

[26] Metscher BD. MicroCT for comparative morphology: simple staining methods allow high-contrast 3D imaging of diverse non-mineralized animal tissues. BMC Physiol. 2009;9:11. doi: 10.1186/1472-6793-9-11 19545439 PMC2717911

[27] Mensa FS, Muzzi M, Spani F, Tromba G, Dullin C, Di Giulio A. When the utility of micro-computed tomography collides with insect sample preparation: an entomologist user guide to solve post-processing issues and achieve optimal 3D models. Appl Sci. 2022;12(2):769. doi: 10.3390/app12020769

[28] Wagner R, Van Loo D, Hossler F, Czymmek K, Pauwels E, Van Hoorebeke L. High-resolution imaging of kidney vascular corrosion casts with Nano-CT. Microsc Microanal. 2011;17(2):215–9. doi: 10.1017/S1431927610094201 21122193

[29] Windfelder AG, Steinbart J, Graser L, Scherberich J, Krombach GA, Vilcinskas A. An enteric ultrastructural surface atlas of the model insect *Manduca sexta*. iScience. 2024;27(4):109410. doi: 10.1016/j.isci.2024.109410 38558941 PMC10981077

[30] Windfelder AG, Steinbart J, Flögel U, Scherberich J, Kampschulte M, Krombach GA, et al. A quantitative micro-tomographic gut atlas of the lepidopteran model insect *Manduca sexta*. iScience. 2023;26(6):106801. doi: 10.1016/j.isci.2023.106801 37378344 PMC10291339

[31] Zemek M, Blažek P, Šrámek J, Salplachta J, Zikmund T, Klapetek P, et al. Voxel size calibration for high-resolution CT. eJNDT. 2020;25(2). doi: 10.58286/25109

[32] Kim I, Paik K-S, Lee S-P. Quantitative evaluation of the accuracy of micro-computed tomography in tooth measurement. Clin Anat. 2007;20(1):27–34. doi: 10.1002/ca.20265 16372341

[33] Guerrero F, Berástegui E. Porosity analysis of MTA and Biodentine cements for use in endodontics by using micro-computed tomography. J Clin Exp Dent. 2018;10(3):e237–40. doi: 10.4317/jced.54688 29721224 PMC5923892

[34] Huang Y, Celikten B, de Faria Vasconcelos K, Ferreira Pinheiro Nicolielo L, Lippiatt N, Buyuksungur A, et al. Micro-CT and nano-CT analysis of filling quality of three different endodontic sealers. Dentomaxillofac Radiol. 2017;46(8):20170223. doi: 10.1259/dmfr.20170223 28845679 PMC5965939

[35] Schoborg TA. Whole Animal Imaging of *Drosophila melanogaster* using Microcomputed Tomography. J Vis Exp. 2020;(163):10.3791/61515. doi: 10.3791/61515 32955492 PMC7891681

[36] Keklikoglou K, Faulwetter S, Chatzinikolaou E, Wils P, Brecko J, Kvaček J, et al. Micro-computed tomography for natural history specimens: a handbook of best practice protocols. EJT. 2019;(522). doi: 10.5852/ejt.2019.522

[37] Hu Z, Gui J, Zou J, Rong J, Zhang Q, Zheng H, et al. Geometric calibration of a micro-CT system and performance for insect imaging. IEEE Trans Inf Technol Biomed. 2011;15(4):655–60. doi: 10.1109/TITB.2011.2159012 21659036

[38] Scherberich J, Windfelder AG, Krombach GA. Analysis of fixation materials in micro-CT: It doesn’t always have to be styrofoam. PLoS One. 2023;18(6):e0286039. doi: 10.1371/journal.pone.0286039 37315002 PMC10266650

[39] Schindelin J, Arganda-Carreras I, Frise E, Kaynig V, Longair M, Pietzsch T, et al. Fiji: an open-source platform for biological-image analysis. Nat Methods. 2012;9(7):676–82. doi: 10.1038/nmeth.2019 22743772 PMC3855844

[40] Hammer Ø, Harper DA, Ryan PD. PAST: paleontological statistics software package for education and data analysis. Palaeont Electr. 2001;4:1–9.

[41] Saxena RC, Friedman S, Bly RA, Otjen J, Alessio AM, Li Y, et al. Comparison of micro-computed tomography and clinical computed tomography protocols for visualization of nasal cartilage before surgical planning for rhinoplasty. JAMA Facial Plast Surg. 2019;21(3):237–43. doi: 10.1001/jamafacial.2018.1931 30730533 PMC6537836

[42] Blažek P, Šrámek J, Zikmund T, Kalasová D, Hortvík V, Klapetek P, et al. Voxel size and calibration for CT measurements with a small field of view. eJNDT. 2019;24(3). doi: 10.58286/23714

[43] Avila RS, Krishnan K, Obuchowski N, Jirapatnakul A, Subramaniam R, Yankelevitz D. Calibration phantom-based prediction of CT lung nodule volume measurement performance. Quant Imaging Med Surg. 2023;13(9):6193–204. doi: 10.21037/qims-22-320 37711774 PMC10498266

[44] Sari NK, Sutanto H, Anam C, Naufal A, Amilia R. The feasibility of cylindrical step-wedge phantom for evaluating modulation transfer function of CT image: variation of field of view. IJSRST. 2023;218–25. doi: 10.32628/ijsrst52310632

