# Supplementary figures and images for: Measurement accuracy of CT systems: The importance of calibration phantoms
Source: PLoS One. 2025 Sep 25;20(9):e0332263. doi: 10.1371/journal.pone.0332263 (PMC12463237; doi:10.1371/journal.pone.0332263)

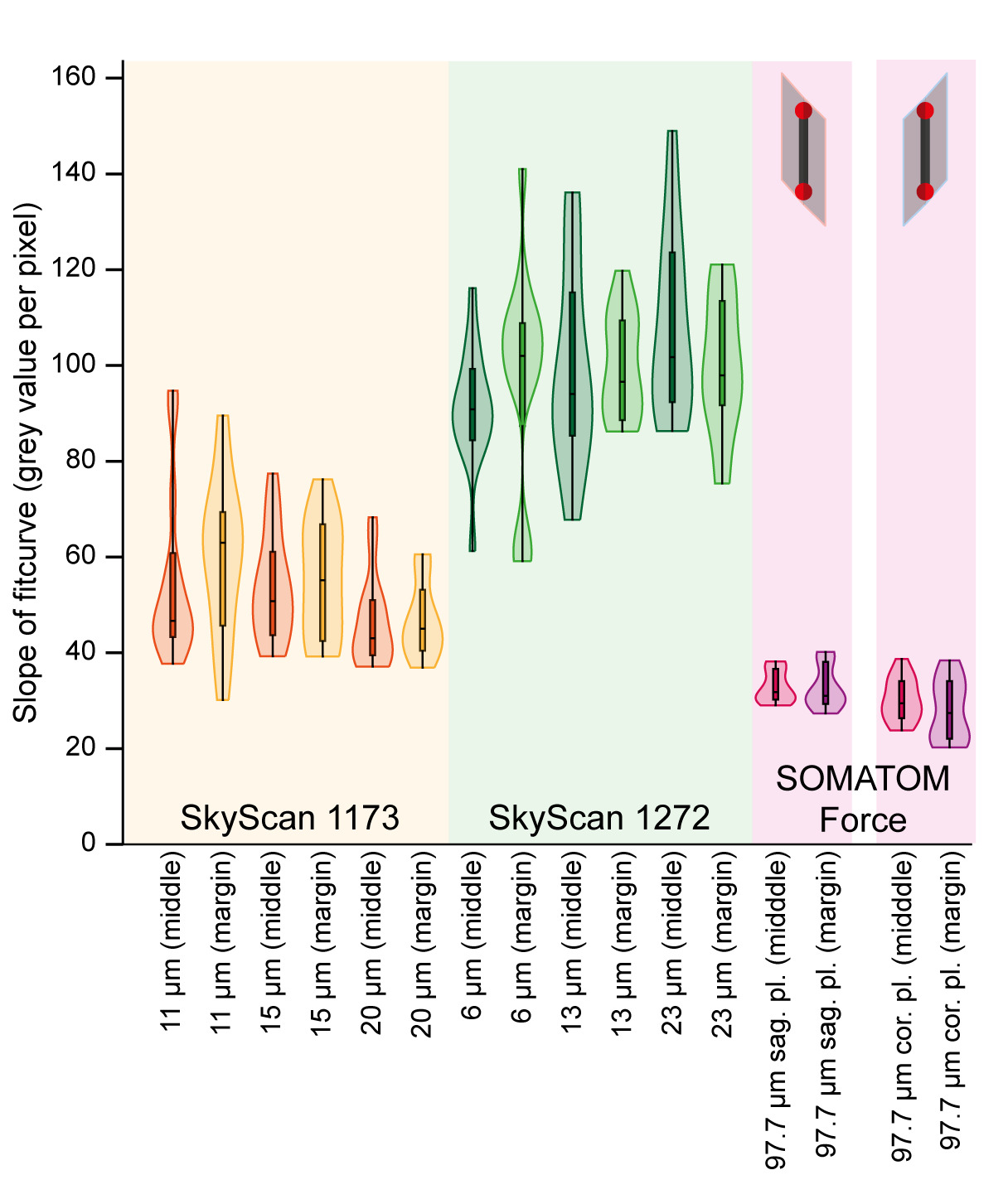

Supplement: S1 Fig — A profile line of 50 pixels (25 pixels for scans of the SOMATOM Force) was placed over the outer edge of the ruby spheres in each CT scan in the 2D view. This was done to determine the gradient of the grey values. The slope of the fit curves (Rodbard fit) was determined for the profile lines and compared with each other. The steepest profile lines were measured in the scans of the high-resolution micro-CT. (TIF) [file pone.0332263.s001.tif]
